# Identification and analysis of lipid metabolism-related genes in allergic rhinitis

**DOI:** 10.1186/s12944-023-01825-z

**Published:** 2023-07-21

**Authors:** Qilei Tao, Yajing Zhu, Tianyu Wang, Yue Deng, Huanhai Liu, Jian Wu

**Affiliations:** Department of Otolaryngology, Changzheng Hospital, Naval Medical University (Second Military Medical University), Shanghai, 200003 China

**Keywords:** Allergic rhinitis, Lipid metabolism, Biomarkers, Diagnostic model, Immune infiltration

## Abstract

**Background:**

Studies have shown that the lipid metabolism mediator leukotriene and prostaglandins are associated with the pathogenesis of allergic rhinitis (AR). The aim of this study was to identify key lipid metabolism-related genes (LMRGs) related to the diagnosis and treatment of AR.

**Materials and methods:**

AR-related expression datasets (GSE75011, GSE46171) were downloaded through the Gene Expression Omnibus (GEO) database. First, weighted gene co-expression network analysis (WGCNA) was used to get AR-related genes (ARRGs). Next, between control and AR groups in GSE75011, differentially expressed genes (DEGs) were screened, and DEGs were intersected with LMRGs to obtain lipid metabolism-related differentially expressed genes (LMR DEGs). Protein-protein interaction (PPI) networks were constructed for these LMR DEGs. Hub genes were then identified through stress, radiality, closeness and edge percolated component (EPC) analysis and intersected with the ARRGs to obtain candidate genes. Biomarkers with diagnostic value were screened via receiver operating characteristic (ROC) curves. Differential immune cells screened between control and AR groups were then assessed for correlation with the diagnostic genes, and clinical correlation analysis and enrichment analysis were performed. Finally, real-time fluorescence quantitative polymerase chain reaction (RT-qPCR) was made on blood samples from control and AR patients to validate these identified diagnostic genes.

**Results:**

73 LMR DEGs were obtained, which were involved in biological processes such as metabolism of lipids and lipid biosynthetic processes. 66 ARRGs and 22 hub genes were intersected to obtain four candidate genes. Three diagnostic genes (LPCAT1, SGPP1, SMARCD3) with diagnostic value were screened according to the AUC > 0.7, with markedly variant between control and AR groups. In addition, two immune cells, regulatory T cells (Treg) and T follicular helper cells (TFH), were marked variations between control and AR groups, and SMARCD3 was significantly associated with TFH. Moreover, SMARCD3 was relevant to immune-related pathways, and correlated significantly with clinical characteristics (age and sex). Finally, RT-qPCR results indicated that changes in the expression of LPCAT1 and SMARCD3 between control and AR groups were consistent with the GSE75011 and GSE46171.

**Conclusion:**

LPCAT1, SGPP1 and SMARCD3 might be used as biomarkers for AR.

**Supplementary Information:**

The online version contains supplementary material available at 10.1186/s12944-023-01825-z.

## Introduction

Allergic rhinitis (AR) is a noninfectious inflammatory disease mediated by IgE that affects approximately 10%~20% of the global population [1]. Exposure to inhaled allergens in susceptible individuals is a frequent precipitating factor for AR. The most common clinical symptoms include paroxysmal sneezing, nasal obstruction, rhinorrhea, and nasal itching, sometimes in association with conjunctivitis, such as eye itching and tearing. Persistent severe rhinitis may predispose patients toward asthma [2]. Moreover, AR patients often have decreased learning and work efficiency, impaired sleep and quality of life, and even psychological disorders such as depression, leading to a huge economic burden on society. For treatment, nasal corticosteroids, antihistamines, and leukotriene receptor antagonists are currently the most recommended drugs [3]. However, their long-term drug use leads to a range of side effects, including epistaxis and drowsiness. Furthermore, the sustained poor efficacy of available drugs causes recurrent illnesses. Thus, it is crucial to find effective therapeutic targets for AR treatment.

Lipids are composed of fats and lipoids and play an important role in different organelles as a second messenger for intracellular signaling [4]. Lipid metabolism refers to the digestion, synthesis, and disassembly of lipids, with the help of various enzymes related to the processing of substances necessary for the body to ensure normal physiologic function. Previous studies have shown that lipid metabolism-related genes (LMRGs) are associated with several systemic diseases. For instance, Li et al. found that LMRGs in circulation have good predictive value for early diagnosis of intervertebral disc degeneration (IDD) [5]. LMRGs are also involved in lung cancer development and might serve as biomarkers for lung cancer [6]. The lipid compound prostaglandins show immunological activity in allergic airway diseases, and meanwhile some prostaglandin D2 (PGD2) receptor antagonists (Ramatroban, AMG 853, etc.) have been shown potentially beneficial effects on allergic inflammation [7]. Leukotriene is a well-recognized lipid inflammatory mediator in allergic diseases, and leukotriene receptor antagonists are one of the major medications for AR [8]. In addition, AR patients have a high level of apolipoprotein in nasal mucus, which may be involved in lipid metabolism and have immunomodulatory properties [9]. As a bioactive lipid, platelet-activating factor (PAF) has the chemotactic property that amplifies mucosal inflammation and causes increased vascular permeability, promoting rhinorrhea and mucus secretion [10]. Peroxisome proliferator-activated receptor gamma (PPAR-γ) may promote activation of type 2 immune response and affect target gene expression by regulating lipid metabolism in allergic diseases [11]. The metabolite of eicosapentaenoic acid (EPA), 15-hydroxyeicosapentaenoic acid (15-HEPE) may reduce allergic rhinitis symptoms via intranasal injection by interacting with PPAR-γ and inhibition of mast cell degranulation [12]. Nevertheless, how LMRGs contribute to AR pathogenesis has remained unclear.

Rapid advances in ‘genomics’ and ‘omics’ yield vast amounts of data and gene-by-gene analysis is insufficient to meet the demands of biological cognition. Compared with laboratory experiments, bioinformatics technology has a great advantage in collating, analyzing, and visualizing large amount of biological information quickly and accurately by computer, with a good reproducibility [13]. Biomarkers and therapeutic targets of many diseases as well as tumors were identified by bioinformatics [14–16]. In this study, AR-related public datasets (GSE75011, GSE46171) were downloaded from Gene Expression Omnibus (GEO) database, and weighted gene co-expression network analysis (WGCNA) was performed to gain AR-related genes (ARRGs). Following the lipid metabolism-related differentially expressed genes (LMR DEGs) were screened, the protein-protein interaction (PPI) networks were constructed for hub genes. Enrichment analysis and receiver operating characteristic (ROC) curve evaluation were subsequently performed to identify diagnostic genes, and single-set gene set enrichment analysis (ssGSEA) was used to analyze the relationship between diagnostic genes and immune cells. Simultaneously, the predictive value of the nomogram based on the diagnostic genes was created and assessed for clinical decision making. This study was aimed to identify potential LMRGs with diagnostic value for AR, providing a potential treatment of AR patients.

## Materials and methods

### Collection of LMRGs

According to the previous literature [17], LMRGs were gained from the Reactome database and Kyoto Encyclopedia of Genes and Genomes (KEGG) database. The LMRG1 within the “Metabolism of lipids” pathway (R-HSA-556,833) located in the Reactome database were extracted and overlapped with the LMRG2 in the 16 lipid metabolism-related gene sets from the KEGG database to obtain 750 LMRGs for subsequently analysis [5].

### Microarray datasets acquisition in AR-related public datasets

Considering the important significance of skin or blood allergy testing in AR diagnosis, the gene expression profiles of GSE75011 from Th2-enriched CD4 + T cells in blood and GSE46171 from nasal epithelial cells within AR samples were selected and downloaded through GEO database. The Illumina HiSeq 2500 sequencing dataset GSE75011, with the largest number of samples was conducted as the training set, containing 15 control and 25 AR blood samples [18]. Besides, GSE46171 dataset containing three control and six AR samples of nasal mucosa was used as an external validation set to enrich disease information and avoid bias among different researches [19].

### Identification of ARRGs

To gain ARRGs in GSE75011, WGCNA was performed [20]. First, the samples were clustered to remove outliers. Thereafter, the determination of soft threshold (β) was performed. Modules were segmented via dynamic tree cutting based on optimal β. Correlations were analyzed between modules and AR. The genes of the highest relevance module with |gene significance (GS)| > 0.3, |module membership (MM)| > 0.6, and *P <* 0.05 were defined as ARRGs [21, 22].

### Screening and functional analysis of LMR DEGs

First, the read count data matrix in GSE75011 was transformed into log-counts per million (log-cpm) value to fit a linear model through voom approach in “limma” R package (version 3.48.3) for differentially expressed analysis. The mRNA expression levels between control and AR groups in the GSE75011 dataset were contrasted with the screening threshold of *P <* 0.05 via the “limma” R package (version 3.48.3) [23]. DEGs and LMRGs were taken to intersect to get LMR DEGs. Subsequently, enrichment analysis for LMR DEGs via Metascape database (*P <* 0.05) [24]. In addition, the online database WebGestalt was used to study the Disease Ontology (DO) function of LMR DEGs [25].

### Creation of the PPI networks of LMR DEGs and screening of hub genes

The PPI network of LMR DEGs were created via Search Tool for the Retrieval of Interacting Genes (STRING) with confidence score more than 0.4 and visualized by Cytoscape. Subsequently, hub genes were further obtained by intersecting the top 30 genes which were calculated and ranked on the basis of among stress, radiality, closeness, and edge percolated component (EPC) within CytoHubba algorithm.

### Screening of diagnostic genes

First, candidate genes were obtained by intersecting hub genes with ARRGs, and enrichment analysis were applied on them. Second, ROC curves of the candidate genes were mapped via “pROC” R package (version 1.18.0) in the GSE75011 and GSE46171 [26]. Candidate genes with area under the curve (AUC) ≥ 0.7 were regarded diagnostic genes. A nomogram was constructed with diagnostic genes for clinical utilize, and a calibration curve of the nomogram was drawn to verify its validity. The diagnostic worth of age, sex, and time point was assessed in GSE46171 via ROC curves.

### Immune analysis

The ssGSEA algorithm was utilized to assess infiltrating richness of immune cells between control and AR groups in the training set. Differences of the control and AR groups were compared by the Wilcox test. In addition, immune cells relevant to the diagnostic genes were detected via Spearman algorithm between diagnostic genes and differential immune cells.

### Analysis of clinical correlation

Relevance between diagnostic genes and clinical characteristics (age, sex, time point) was analyzed using Pearson correlation analysis in the “corrplot” R package [27].

### Gene Set Enrichment Analysis (GSEA) of diagnostic genes

On the basis of the median value of the diagnostic gene expressions, the samples of GSE75011 were grouped into high and low expression groups. All genes in two expression groups were performed GSEA with |normalized enrichment score (NES)| > 1, nominal (NOM) *P* value < 0.05, and *q* < 0.25 [28].

### Expression profiles of diagnostic genes in external validation datasets

To further demonstrate the reliability of the results above, expression levels of the diagnostic genes between control and AR samples were compared in the GSE75011 and GSE46171 datasets for external validation.

### Patients and tissue preparation

Ten AR patients and ten patients without AR or significant underlying disease were selected from people visiting to Shanghai Changzheng Hospital. There were no marked variation in sex and age between the groups (Table 1). Blood samples were acquired from these patients with informed consent and carried out real-time fluorescence quantitative polymerase chain reaction (RT-qPCR). The Medical Ethics Committee of Shanghai Changzheng Hospital endorsed this study.

**Table 1:** Basic information of the patients

Group		Sex		Age (mean ± STD)
		Male	Female	
AR		6	4	29.9 ± 5.705
Control		5	5	28.4 ± 3.098

STD, standard deviation

### RT‒qPCR

Firstly, we conducted the total RNA extraction utilizing TRIzol (Ambion, Austin, USA). Then, reverse transcription of total RNA to cDNA was made via First-strand-cDNA-synthesis-kit (Servicebio, Wuhan, China). RT-qPCR was made utilizing the 2xUniversal Blue SYBR Green qPCR Master Mix (Servicebio, Wuhan, China). Specific experimental steps were carried out on the basis of instructions. The primer sequences were showcased in Additional file 1. Internal reference gene was GAPDH. The 2^−ΔΔCt^ method was utilized to calculate the expression of diagnostic genes [29]. Levels of expression of diagnostic genes between the control and AR groups were compared by the T test.

### Statistical analysis

Statistical analysis was carried out through GraphPad Prism 5 and R software (version 4.2.0). *P* < 0.05 represented a significant difference. Differences between groups were analyzed via the Wilcoxon test.

## Results

### Acquisition of ARRGs

**Fig. 1:**
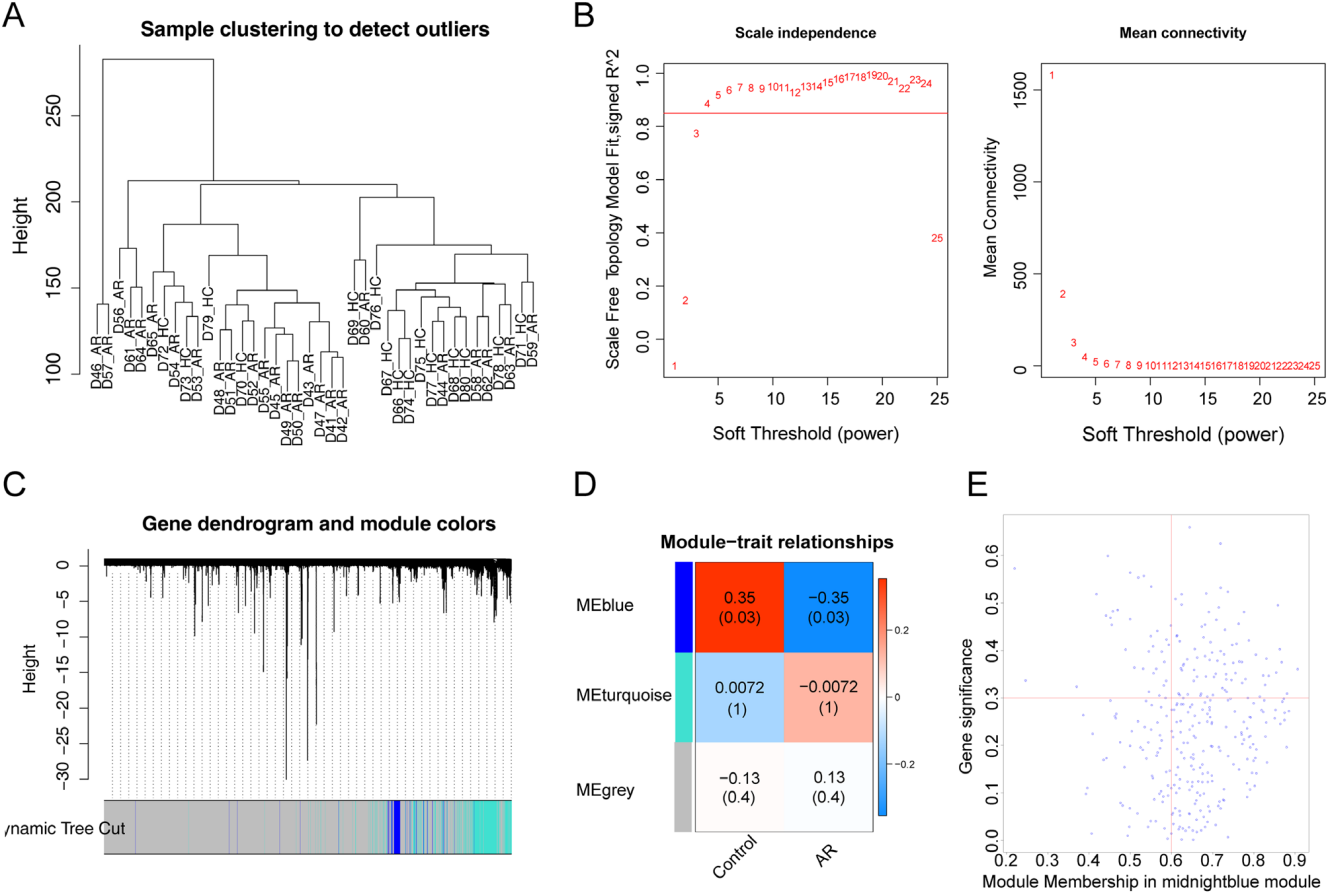
**Gene co-expression network of AR.** (A) Sample clustering analysis of the GSE75011 dataset. (B) Analysis of network topology for various soft-thresholding powers showed that the soft threshold was 4. (C) Clustering dendrograms and modules identified by WGCNA, where the minimum gene number was 100. (D) Module-trait relationships demonstrated that the blue module correlated markedly with AR (cor = -0.35, *P* = 0.03) in three modules, and each square contains the corresponding correlation and *P* value. (E) Correlation scatterplot of 66 ARRGs with |GS| > 0.3, |MM| > 0.6, and *P <* 0.05 in the blue module

To identify ARRGs, WGCNA was performed with the GSE75011 dataset. Sample clustering analysis showed no outliers in the dataset (Fig. 1A). The β was 4 (Fig. 1B), and each gene module contained a minimum of 100 genes. Three modules were eventually identified, each with a unique color (Fig. 1C-D). The blue module correlated markedly with AR (cor = -0.35, *P* = 0.03) (Fig. 1D). Finally, 66 ARRGs were gained and utilized for further analysis (Fig. 1E).

## Acquisition and functional enrichment of LMR DEGs

**Fig. 2:**
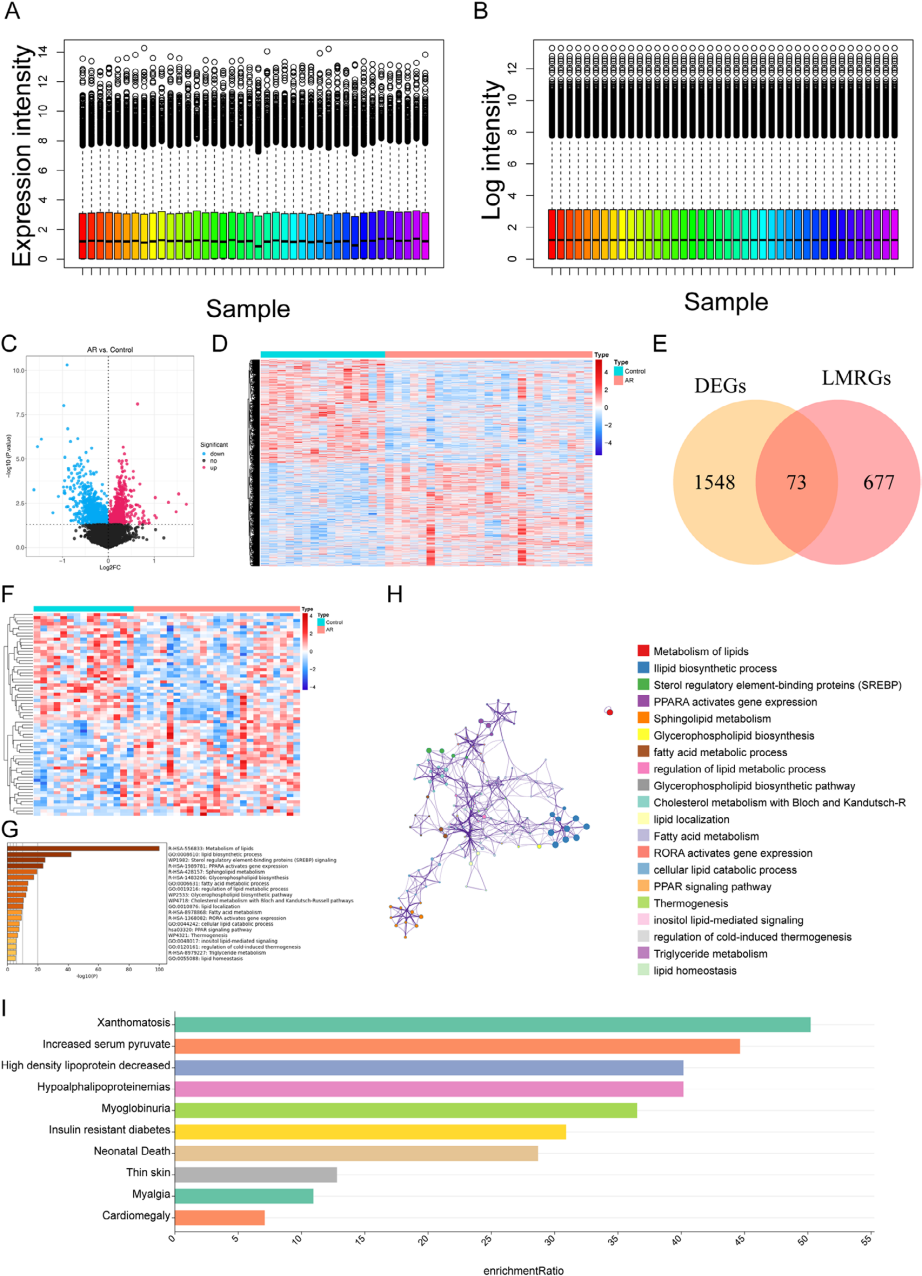
**Acquisition and analysis of LMR DEGs.** (A-B) The 25 samples of the GSE75011 dataset were standardized and presented as box plots. (C) Volcano plot of 1621 DEGs between the AR and control groups (*P <* 0.05). (D) Heatmap of 1621 DEGs between the AR and control groups (*P <* 0.05). (E) Venn diagram of 73 LMR DEGs by overlapping LMRGs and DEGs. (F) Heatmap of the 73 LMR DEGs. (G-H) Enrichment analysis of the 73 LMR DEGs in Metascape was performed, and the associated interaction network is shown. (I) DO function analysis of LMR DEGs using the WebGestalt database

The 25 samples were standardized for the GSE75011 dataset and are presented as box plots in Fig. 2A-B. The volcano plot and heatmap show 1621 DEGs between the AR and control groups, including 810 upregulated and 811 downregulated genes (Fig. 2C-D). A total of 73 LMR DEGs (Additional file 2) were obtained by Venn analysis with LMRGs (750 genes) and DEGs (1621 genes), with a significant difference detected based on a heatmap (Fig. 2E-F). Enrichment analysis of the 73 LMR DEGs by Metascape showed a total of 334 functional pathways (Fig. 2G-H) to be related to the LMR DEGs, such as metabolism of lipids, lipid biosynthetic process, and sterol regulatory element-binding protein (SREBP) signaling. DO enrichment results showed that the LMR DEGs are significantly associated with 10 diseases, namely, xanthomatosis, increased serum pyruvate, decreased high-density lipoprotein, hypoalphalipoproteinemia, myoglobinuria, insulin-resistant diabetes, neonatal death, thin skin, myalgia and cardiomegaly (Fig. 2I).

### Acquisition of hub genes

**Fig. 3:**
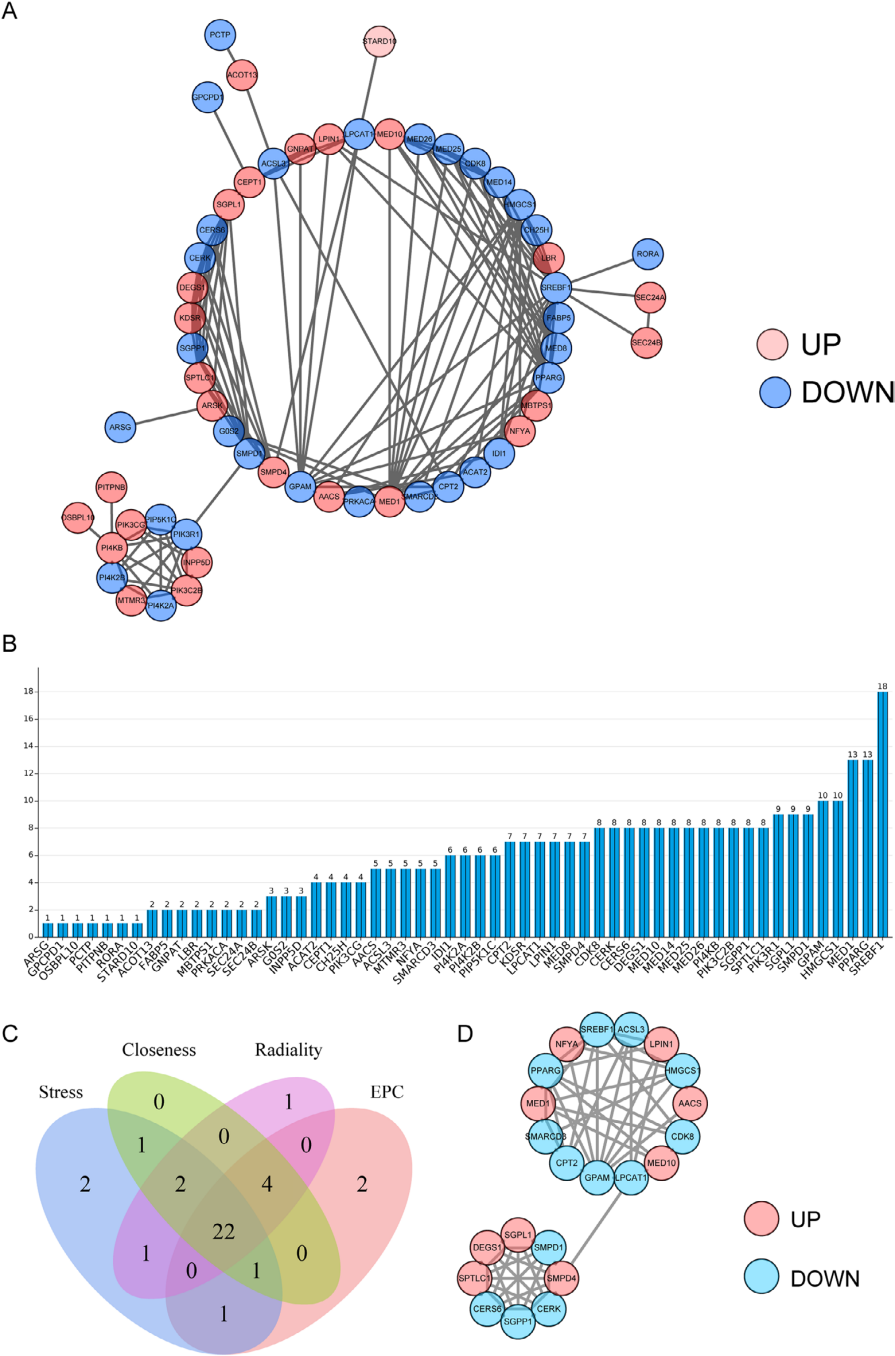
**Acquisition of hub genes.** (A) The protein-protein interaction (PPI) network for 73 LMR DEGs through STRING. (B) The degree of connectivity of each gene in the PPI network demonstrated that SREBF1 interacted with multiple proteins. (C) Venn diagram of hub genes common to four algorithms. (D) PPI network of hub genes

The PPI network was created for LMR DEGs. As illustrated in Fig. 3A-B, SREBF1 interacts with multiple proteins, such as LPIN1, GPAM, and MED1. To identify the most important genes, the 22 genes common to the four algorithms were used as hub genes (Fig. 3C), and a PPI network of hub genes was created (Fig. 3D). The results showed that GPAM interacts with seven genes, namely, PPARG, NFYA, SREBF1, ACSL3, LPIN1, HMGCS1 and AACS.

### Acquisition of diagnostic genes

**Fig. 4:**
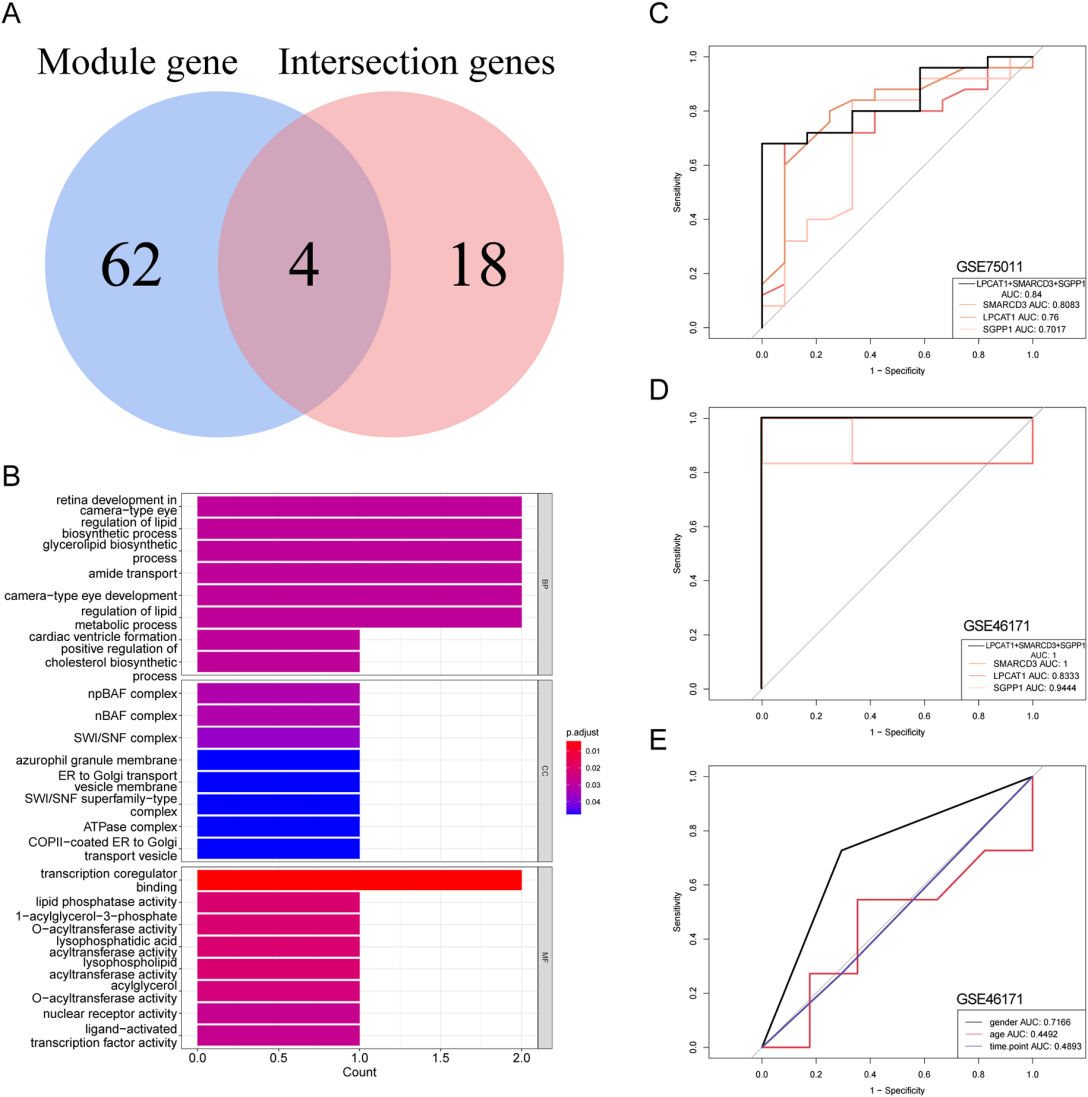
**Acquisition of diagnostic genes.** (A) Venn diagram of four candidate genes by taking the intersections of ARRGs and hub genes. (B) Gene Ontology (GO) enrichment analysis of the four candidate genes. (C-D) AUC curves of the three genes for diagnostic prediction in the GSE75011 and GSE46171 datasets. (E) AUCs of age, sex, and time point in GSE46171

Four candidate genes associated with diagnosis of AR were obtained by 66 ARRGs with 22 hub genes taking intersections: LPCAT1, SREBF1, SMARCD3, and SGPP1 (Fig. 4A). The four candidate genes were involved in 133 Gene Ontology (GO) items, including 114 GO biological process (BP), nine GO cellular component (CC) and 10 GO molecular function (MF), such as retina development in camera-type eye, npBAF complex, and transcription coregulator binding (Fig. 4B).

The diagnostic value of four candidate genes was assessed via ROC curve in GSE75011 and GSE46171. The AUC values for the three genes (LPCAT1, SMARCD3, and SGPP1) were greater than 0.7 in both datasets, suggesting that the three genes have diagnostic value for AR (Fig. 4C-D). The AUCs for age, sex and time point were 0.4492, 0.7166, and 0.4893, respectively, in GSE46171, revealing that sex might be a diagnostic factor for AR (Fig. 4E).

**Fig. 5:**
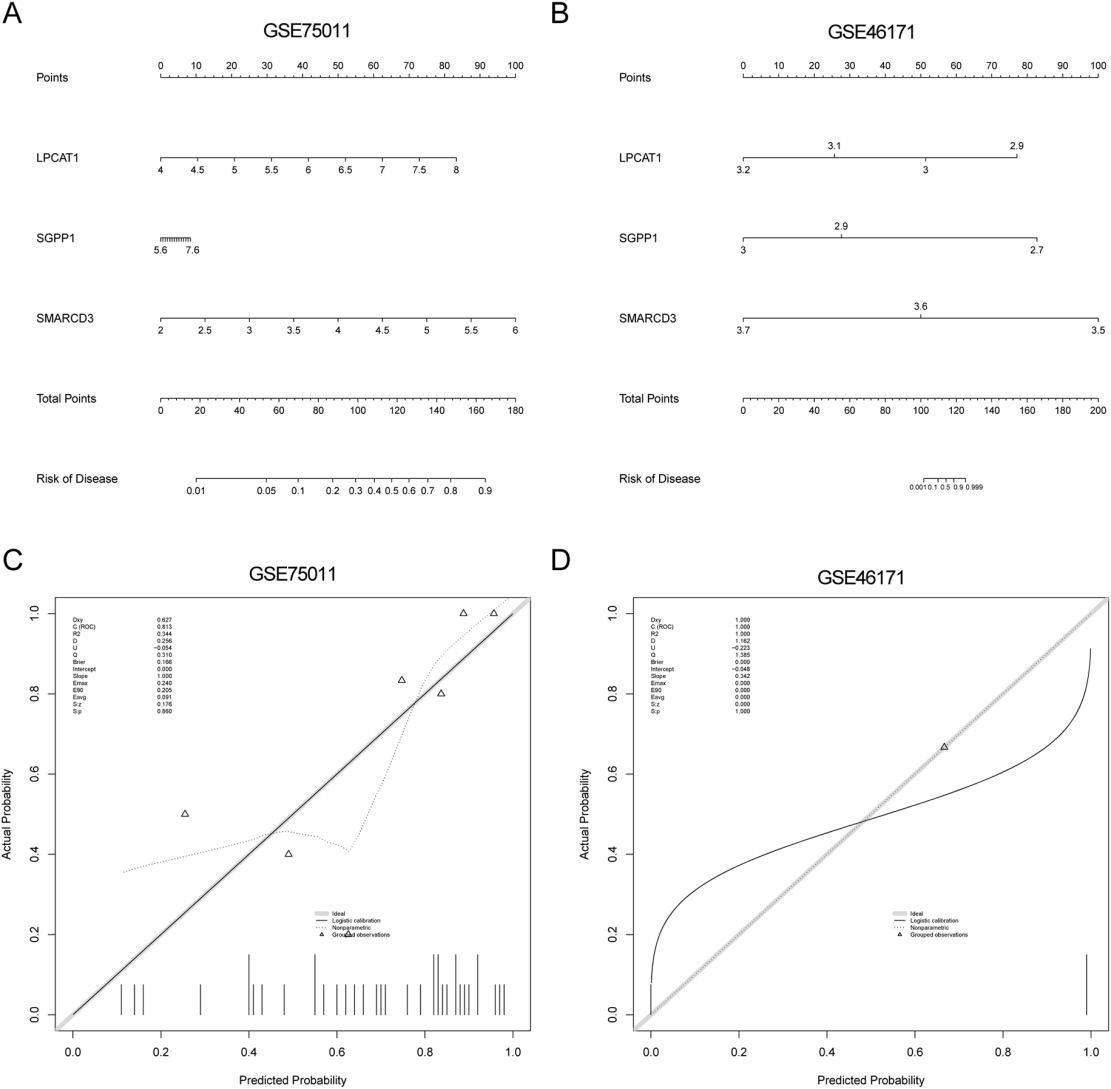
(A-B) The nomogram drawn based on the three diagnostic genes for the diagnostic efficacy of AR in GSE75011 and GSE46171. (C-D) Calibration curves of the nomogram in GSE75011 and GSE46171

Finally, the nomograms were created containing the three diagnostic genes in GSE75011 and GSE46171 (Fig. 5A-B), and the AUC values in both datasets were above 0.6 (Fig. 5C-D). The results demonstrated that the nomogram has good prediction ability for AR.

### Immuno-infiltration analysis in control and AR groups

**Fig. 6:**
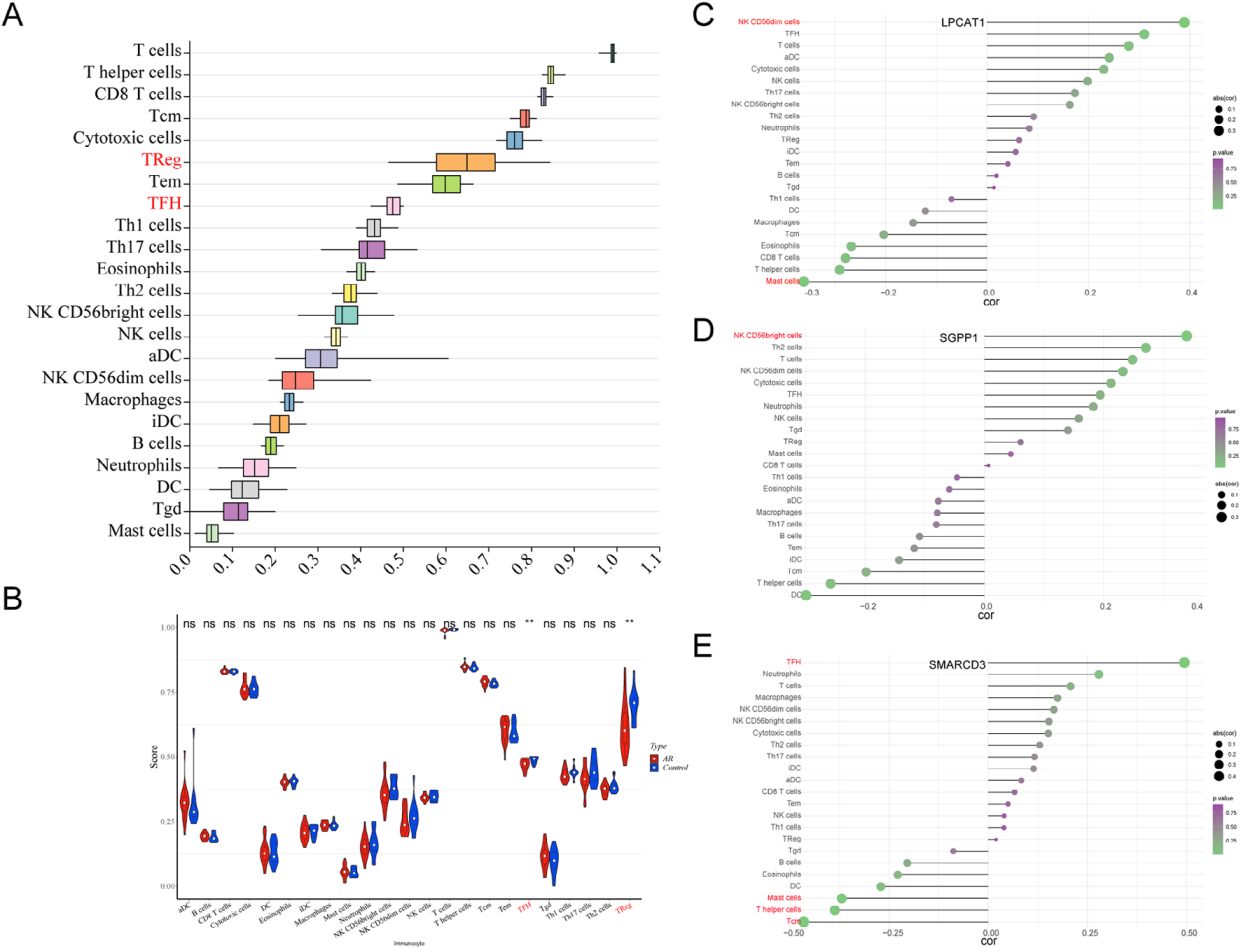
**Immuno-infiltration analysis in AR.** (A) Analysis of the percentage of immune cells in all samples of GSE75011 using ssGESA. (B) Differences in immune cell infiltration between control and AR groups (Wilcox. Test). **P < 0.01. Lollipop chart demonstrating the correlation between diagnostic genes and immune cells, including (C) LPCAT, (D) SGPP1, and (E) SMARCD3

Analysis of the percentage of immune cells by ssGESA in all samples showed the highest for T cells (Fig. 6A). Differences in infiltrating immune cells between the control and AR groups were illustrated by a violin plot (Fig. 6B). The results suggested that infiltration of regulatory T cells (Tregs) and T follicular helper cells (TFHs) was markedly lower in the AR group. There was significant relevance between SMARCD3 and TFHs. However, neither LPCAT1 nor SGPP1 correlated with differential immune cells (Tregs and TFHs); therefore, SMARCD3 was selected for further analysis (Fig. 6C-E).

### Correlation analysis of clinical features, enrichment analysis and infiltration analysis of SMARCD3

**Fig. 7:**
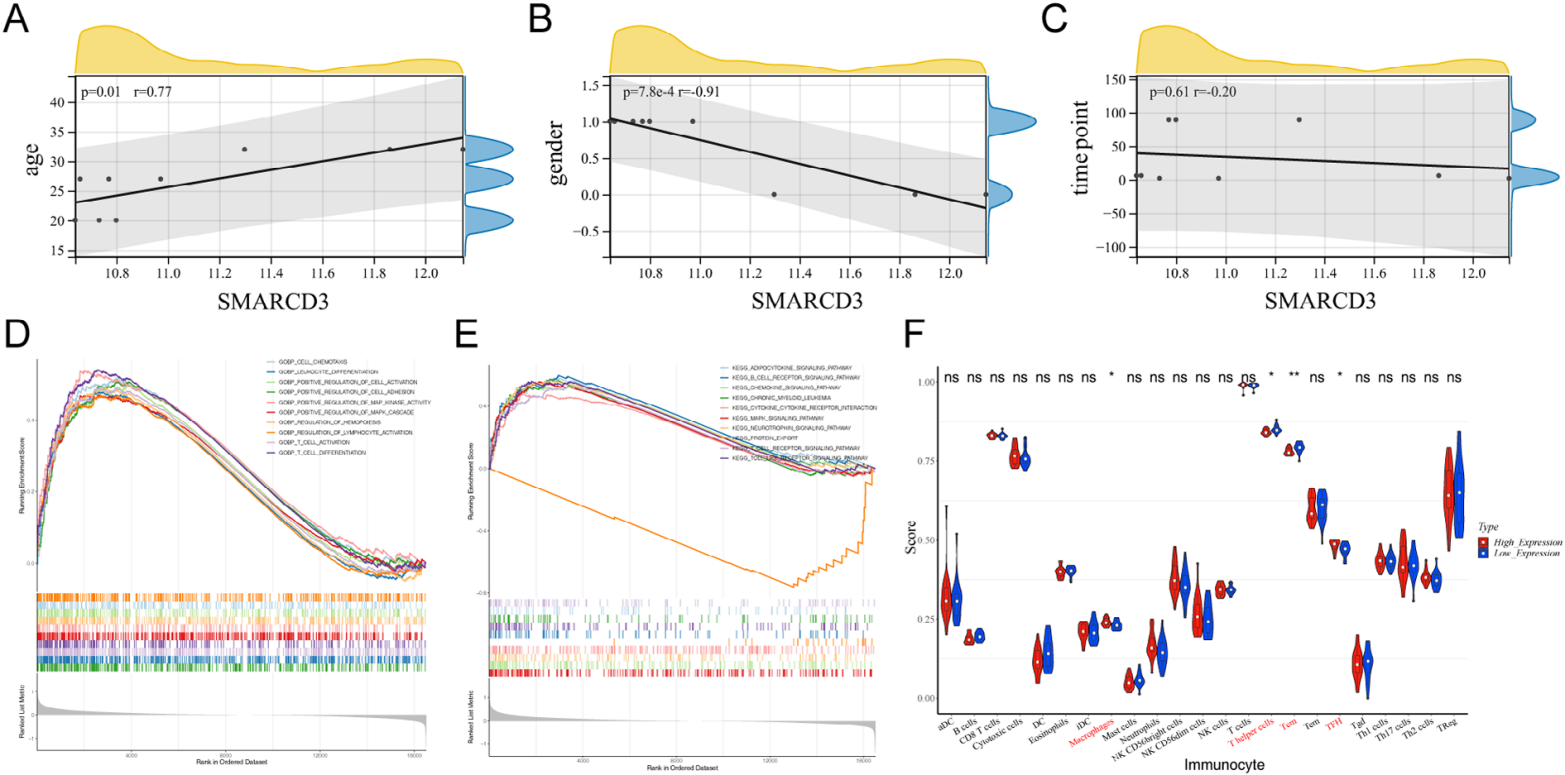
**Comprehensive analysis of SMARCD3 in AR.** (A-C) Pearson correlation analysis of SMARCD3 clinical characteristics (age, sex and time point). GSEA of SMARCD3 showed that 256 GO enrichment pathways (D) and 33 KEGG pathways (E) were enriched in AR. (F) Differences in immune cell infiltration between high and low SMARCD3 expression groups (Wilcox test). *P < 0.05; **P < 0.01

Pearson correlation analysis demonstrated that SMARCD3 was significantly associated with clinical characteristics (age and sex) (Fig. 7A-C). Then, GSEA for SMARCD3 was performed, revealing 256 GO enrichment (Additional file 3) and 33 KEGG pathways (Additional file 4) (Fig. 7D-E). Overall, SMARCD3 was involved in immune-related pathways, for instance, the B-cell receptor signaling pathway and T-cell receptor signaling pathway. Four immune cells displayed marked variations between the high and low expression groups, namely, macrophages, T helper cells, central memory T cell (Tcm), and TFH cells, reflecting the strong relevance between SMARCD3 and the immune microenvironment (Fig. 7F).

### mRNA levels of diagnostic genes

**Fig. 8:**
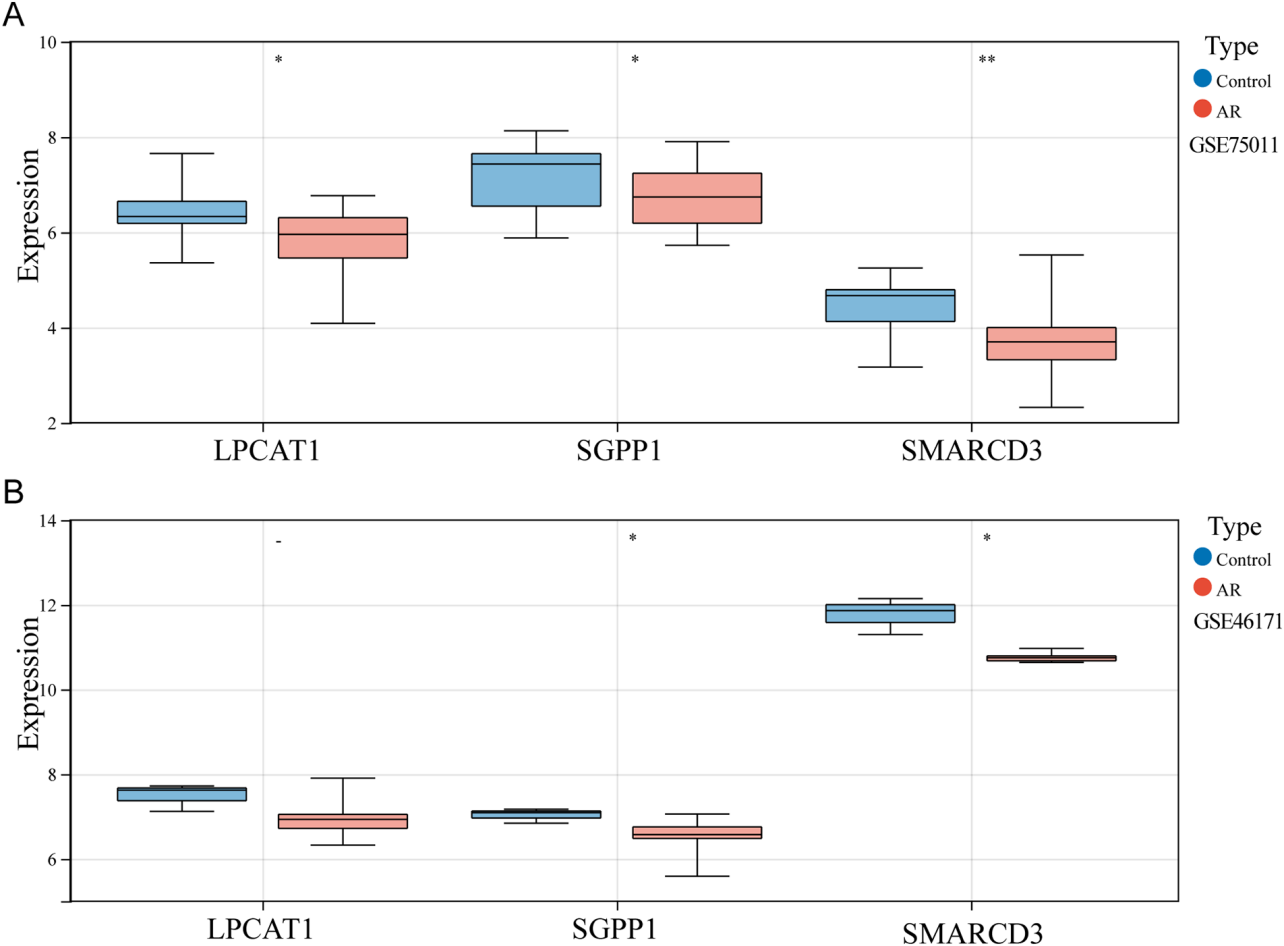
**Expression levels of three diagnostic genes in online datasets.** (A) GSE75011. (B) GSE46171. *P < 0.05; **P < 0.01

The significant differences in expression of SGPP1, LPCAT1 and SMARCD3 between control and AR in GSE75011 and GSE46171 were clearly observed via visualized data (Fig. 8A-B). Moreover, the changes of the three genes expression were consistent in blood and nasal mucosal tissues, suggesting that these three genes are of high diagnostic value.

**Fig. 9:**
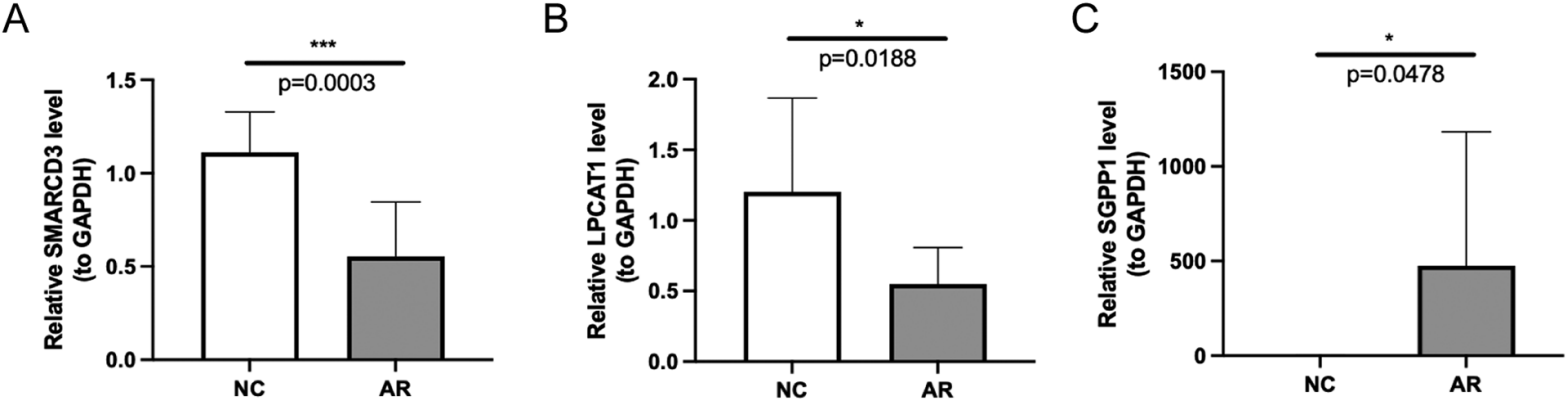
**Validation of diagnostic genes expression by RT-qPCR.** (A) SMARCD3. (B) LPCAT1. (C) SGPP1. *P < 0.05; ***P < 0.001

To verify the three diagnostic genes expression, we collected blood samples to assess mRNA expression levels of these genes via RT-qPCR. The expression trends of LPCAT1 and SMARCD3 were consistent with public databases, and the expression was lower in AR group (Fig. 9A-B). However, SGPP1 exhibited the opposite trend compared to the results of public database, possibly due to different experimental designs or analysis methods (Fig. 9C).

## Discussion

AR is an airway allergic disease with a high incidence, affecting billions of people in the world. Nevertheless, the effect of current therapies for AR is unsatisfactory due to its complex pathogenesis. LMRGs are involved in the maintenance of systemic physiology and play an important role in diverse diseases, especially in malignant tumors. Moreover, lipid-related inflammatory mediators such as prostaglandins and leukotrienes have been implicated in AR pathogenesis. To our knowledge, this is the first study to identify and analyze LMRGs in AR.

In this study, three key LMRGs most associated with AR, i.e., LPCAT1, SGPP1, and SMARCD3, were identified, all of which are protein-coding genes. As one of the lysophosphatidylcholine acyltransferase (LPCAT) family, the LPCAT1 protein is an enzyme essential for phosphatidylcholine metabolism and regulation of phosphatidylcholine composition [30]. LPCAT1 is also used in the prognosis of multiple tumors, such as breast cancer, colorectal cancer, and hepatocellular carcinoma [31–33]. Little is known about LPCAT1 in allergic diseases. One study reported that LPCAT1 downregulates eosinophilic inflammation in asthmatic mice [34]. In the current study, LPCAT1 was significantly lower in AR blood samples, consistent with published results, suggesting that it may be essential for AR pathogenesis. SGPP1 can catalyze degradation of S1P, who can regulate diverse biological processes, as a bioactive sphingolipid metabolite [35]. SGPP1 is considered to be closely related to several tumors, especially regarding chemoresistance and radioresistance [36, 37]. There are currently no reports about the function of SGPP1 in allergic diseases, and the results in the current study are the first to show significant downregulation of SGPP1 in both blood and nasal mucosa samples in AR patients; conversely, RT-qPCR using blood samples showed the opposite result, possibly due to different experimental designs or analysis methods. Thus, the effect of SGPP1 in AR is still unclear. SMARCD3 is a chromatin-remodeling factor and a member of the SWI/SNF family, which present helicase and ATPase activities and are crucial in the transcription process of certain genes. Its related pathways include the circadian clock and transcriptional activation of mitochondrial biogenesis [38]. SMARCD3 was found to be downregulated in AR patients in this study, but how it participates in disease processes remains to be explored.

Immuno-infiltration analysis refers to studying the composition and quantification of immune cells in diseases. In this study, TFHs were extremely significantly reduced in the AR group. TFHs are CD4^+^ T cells that specialize in helping B cells and are involved in a wide range of diseases. An increasing number of theories have concluded that the antigen-related IgE response depends on more TFHs than Th2 cells [39, 40]. There are few reports about TFH and SMARCD3. A microarray model system identified that the SMARCD3 gene is upregulated in T-cell acute lymphoblastic leukemia [41]. In this study, only SMARCD3 correlated with differential immune cells (Tregs and TFHs), and TFHs and SMARCD3 were downregulated simultaneously in AR patients. Hence, it is hypothesized that SMARCD3 participates in the differentiation of T cells.

GSEA was performed to further investigate the role of SMARCD3 in AR, the results of which showed significant enrichment in the adipocytokine signaling pathway, B-cell receptor signaling pathway, and chemokine signaling pathway, among others. The adipocytokine signaling pathway refers to a series of cascade events via autocrine or paracrine adipocytokines, such as leptin and adiponectin, by adipocytes in the body [42, 43]. This pathway is not only crucial for obesity, insulin resistance, and type 2 diabetes mellitus but also plays an important role in inflammation and allergic diseases. Dysregulation of pulmonary adipocytokine/insulin signaling caused by early-onset obesity has been proven to induce asthma-like disease in mice [44]. The leptin/osteopontin axis promotes Th2 inflammation and Th17 responses in AR through the NF-кB, MAPK, JNK pathway and β3 integrin [45, 46]. Signaling through the B-cell receptor (BCR) is crucial for antigen recognition and subsequent biological effects, including B-cell activation, proliferation, and differentiation, which ensure host defense [47]. One study demonstrated that the BCR signaling pathway was significantly enriched among differentially expressed vesicle miRNAs in AR patient nasal mucus, consistent with the findings in the current study and further elucidating the importance of the BCR signaling pathway in AR development [48]. Chemokines are small molecule-scale cytokines that recruit leukocyte subsets under steady-state and pathological conditions; signaling pathways are activated by their binding to receptors on the cell surface and are involved in chronic inflammatory and autoimmune diseases. Multiple studies have shown that knockdown of the chemokine receptor CCR3 reduces eosinophilic inflammation and the Th2 immune response in AR [49–51]. In summary, our findings are in accordance with all of the above studies.

### Comparisons with other studies and contribution of the current work to existing knowledge

To the best of our knowledge, previous exploration of AR based on GSE75011 and GSE46171 has mainly targeted key genes differentially expressed between control and AR samples [18, 19, 52, 53]. In the current study, the biological significance of lipid metabolism in AR was first systematically explored at the genetic level through these same datasets. Moreover, immune infiltration analysis was conducted to investigate the immune cell targets associated with SMARCD3 to elucidate the underlying role of immune-related treatment targeting the SMARCD3 gene in exploration of AR development.

### Study strengths and limitations

Three key LMRGs with high diagnostic value for AR were identified and analyzed for the first time based on bioinformatics analysis of AR-related expression datasets. However, the limitations of this study cannot be ignored. First, small sample sizes and small datasets of AR may have introduced bias. Second, the mechanisms of these genes in AR development have not been clearly elucidated. Further research is needed for the possibility of clinical use in the future.

## Conclusions

In summary, this is the first bioinformatics analysis of LMRGs in AR, and three key genes (LPCAT1, SGPP1 and SMARCD3) with high diagnostic value for AR were identified, where the expression of two of these genes were confirmed by clinical validation and are considered potential treatment targets. Not only the predictive capability of these key LMRGs were confirmed more excellent than that of other clinical features both in the GSE75011 and GSE46171, but also the nomogram by converting the expression of the key LMRGs into a score was constructed to take all of which into consideration for clinical utilize. Considering the samples regarding the AR datasets used in the study were all from peripheral blood, we make the case that the nomogram could be considered as an effectively clinical diagnostic and practical device with the help of the peripheral blood tests and evaluation of the key LMRGs expressions. Besides, correlation of SMARCD3 expression and immune cell infiltration was helpful to reveal future research directions of immune-related treatment targeting the SMARCD3 gene in AR.

### Electronic supplementary material

Below is the link to the electronic supplementary material.

Supplementary Materials 1: Table S1. Primer sequences for real-time fluorescence quantitative polymerase chain reaction (RT-qPCR)

Supplementary Materials 2: Table S2. List of 73 lipid metabolism-related differentially expressed genes (LMR DEGs)

Supplementary Materials 3: Table S3. Results of the 256 Gene Ontology (GO) enriched terms using gene set enrichment analysis (GSEA)

Supplementary Materials 4: Table S4. Results of the 33 Kyoto Encyclopedia of Genes and Genomes (KEGG) enriched terms using GSEA

Supplementary Materials 5: iThenticate repor

Supplementary Materials 6: SNAS Editing Certificate

## Data Availability

GSE75011 and GSE46171 were downloaded from the GEO database (https://www.ncbi.nlm.nih.gov/).

## Abbreviations

AR: allergic rhinitis
LMRGs: lipid metabolism-related genes
GEO: Gene Expression Omnibus
WGCNA: weighted gene co-expression network analysis
ARRGs: AR-related genes
DEGs: differentially expressed genes
PPI: protein-protein interaction
EPC: edge percolated component
ROC: receiver operating characteristic
RT-qPCR: real-time fluorescence quantitative polymerase chain reaction
IDD: intervertebral disc degeneration
PGD2: prostaglandin D2
PAF: platelet-activating factor
PPAR-γ: peroxisome proliferator-activated receptor gamma
EPA: eicosapentaenoic acid
15-HEPE: 15-hydroxyeicosapentaenoic acid
KEGG: Kyoto Encyclopedia of Genes and Genomes
TOM: topological overlap matrix
GS: gene significance
MM: module membership
GO: Gene Ontology
DO: Disease Ontology
STRING: Search Tool for the Retrieval of Interacting Genes
AUC: area under the curve
ssGSEA: single-set gene set enrichment analysis
GSEA: gene set enrichment analysis
NES: normalized enrichment score
NOM: nominal
GAPDH: glyceraldehyde-3-phosphate dehydrogenase
SREBP: sterol regulatory element-binding protein
BP: biological process
CC: cellular component
MF: molecular function
Treg: regulatory T cell
TFH: T follicular helper cell
Tcm: central memory T cell
BCR: B-cell receptor
