# Correction to: Identification and analysis of lipid metabolism-related genes in allergic rhinitis

**DOI:** 10.1186/s12944-023-01893-1

**Published:** 2023-08-04

**Authors:** Qilei Tao, Yajing Zhu, Tianyu Wang, Yue Deng, Huanhai Liu, Jian Wu

**Affiliations:** Department of Otolaryngology, Changzheng Hospital, Naval Medical University (Second Military Medical University), Shanghai, 200003 China

**Correction: Lipids in Health and Disease 22, 105 (2023)**

10.1186/s12944-023-01825-z

Following publication of the original article [1], the authors noticed that the equal contributor symbol to authors “Qilei Tao” and “Yajing Zhu” were missing.

The original article [1] has been updated.

## References

[1] Tao Q, Zhu Y, Wang T (2023). Identification and analysis of lipid metabolism-related genes in allergic rhinitis. Lipids Health Dis.

