# Supplementary material for: Identification and analysis of lipid metabolism-related genes in allergic rhinitis
Source: Lipids Health Dis. 2023 Jul 21;22:105. doi: 10.1186/s12944-023-01825-z (PMC10362667; doi:10.1186/s12944-023-01825-z)
Supplement: Supplementary file 5 — Supplementary Materials 5: iThenticate repor [file 12944_2023_1825_MOESM5_ESM.pdf]

PAPER NAME

Identification and analysis of lipid metab olism-related genes in allergic rhinitis

WORD COUNT

4593 Words

CHARACTER COUNT

26282 Characters

PAGE COUNT

19 Pages

FILE SIZE

125.3KB

SUBMISSION DATE

Jan 10, 2023 2:51 PM GMT+8

REPORT DATE

Jan 10, 2023 2:52 PM GMT+8

13% Overall Similarity

The combined total of all matches, including overlapping sources, for each database.

- 9% Internet database
- 10% Publications database
- Crossref database
- Crossref Posted Content database
- 0% Submitted Works database

Excluded from Similarity Report

- Bibliographic material

Identification and analysis of lipid metabolism-related genes in allergic rhinitis

ABSTRACT

Background: Studies have shown that the lipid metabolism mediator leukotriene is associated with the pathogenesis of allergic rhinitis (AR). The purpose of this study was to demonstrate the key lipid metabolism-related genes (LMRGs) related to the diagnosis and treatment of AR.

Material and methods: AR-related expression datasets (GSE75011, GSE46171) were downloaded through the Gene Expression Omnibus (GEO) database. First, weighted gene coexpression network analysis (WGCNA) was used to get AR-related genes (ARRGs). Next, between control and AR guoups in GSE75011, differentially expressed genes (DEGs) were screened, and DEGs were intersected with LMRGs to obtain lipid metabolism-related differentially expressed genes (LMR DEGs). Protein–protein interaction (PPI) networks were constructed for these LMR DEGs. Hub genes were then identified through stress, radiality, closeness and edge percolated component (EPC) analysis and intersected with the ARRGs to obtain candidate genes. Biomarkers with diagnostic value were screened via receiver operating characteristic (ROC) curves. Differential immune cells screened between control and AR groups were then assessed for correlation with the diagnostic genes, and clinical correlation analysis and enrichment analysis were performed. Finally, reverse transcription-polymerase chain reaction (RT–qPCR) was made on blood samples from

control and AR patients to validate these identified diagnostic genes.

Results: 73 LMR DEGs were obtained, which were involved in biological processes such as metabolism of lipids and lipid biosynthetic processes. Sixty-six ARRGs and 22 hub genes were intersected to obtain four candidate genes. Three diagnostic genes (LPCAT1, SGPP1, SMARCD3) with diagnostic value were screened according to the AUC > 0.7, with markedly variant between control and AR groups. In addition, two immune cells, regulatory T cells (TReg) and T follicular helper cells (TFH), were marked variations between control and AR groups, and SMARCD3 was significantly associated with TFH. Moreover, SMARCD3 was relevant to immue-related pathways, and correlated significantly with clinical characteristics (age and sex). Finally, RT–qPCR results indicated that changes in the expression of LPCAT1 and SMARCD3 between control and AR groups were consistent with the GSE75011 and GSE46171.

Conclusion: LPCAT1, SGPP1 and SMARCD3 might be used as biomarkers for AR.

Keywords: Allergic rhinitis; Lipid metabolism; Biomarkers; Diagnostic model; Immune infiltration

Introduction

Allergic rhinitis (AR) is a chronic atopic disease characterized by the generation of specific IgE that affects approximately 10%~20% of the global population [1]. Exposure to inhaled allergens in susceptible individuals is a frequent precipitating factor for AR. The most common clinical symptoms include paroxysmal sneezing, nasal obstruction, rhinorrhea, and nasal itching, sometimes in association with

conjunctivitis, such as eye itching and tearing. Persistent severe rhinitis may predispose patients toward asthma [2]. Moreover, AR patients often have decreased learning and work efficiency, impaired sleep and quality of life, and even psychological disorders such as depression, leading to a huge economic burden on society. For treatment, nasal corticosteroids, antihistamines, and leukotriene receptor antagonists are currently the most recommended drugs [3]. However, their long-term drug use leads to a range of side effects, including epistaxis and drowsiness. Furthermore, the sustained poor efficacy of available drugs causes recurrent illnesses. Thus, finding more effective treatment for AR treatment is crucial.

Lipids are composed of fats and lipoids and play an important role in different organelles as a second messenger for intracellular signaling [4]. Lipid metabolism refers to the digestion, synthesis, and disassembly of lipids, with the help of various enzymes related to the processing of substances necessary for the body to ensure normal physiologic function. Previous studies showed that lipid metabolism-related genes (LMRGs) are associated with several systemic diseases. For instance, Li et al. found that LMRGs in circulation have good predictive value for early diagnosis of intervertebral disc degeneration (IDD) [5]. LMRGs are also involved in lung cancer development and might serve as biomarkers for lung cancer [6], and the lipid compound prostaglandins is an effective therapeutic target in allergic airway diseases [7]. Leukotriene is a well-recognized lipid inflammatory mediator in allergic diseases, and leukotriene receptor antagonists are one of the major medications for AR [8]. In addition, AR patients have a high level of apolipoprotein in nasal mucus, which may

be involved in lipid metabolism and have immunomodulatory properties [9]. Nevertheless, the relationship between LMRGs and AR has remained unclear.

In this study, AR-related public datasets and comprehensive bioinformatics methods were used to identify LMRGs with diagnostic value for AR, providing a potential treatment of AR patients.

Materials and methods

Data extraction

AR-related datasets (GSE75011 and GSE46171) were downloaded through GEO database. The GSE75011 was the training set, containing 15 control and 25 AR blood samples. GSE46171 dataset containing 3 control and 6 AR samples of nasal mucous was used as an external validation set. A total of 750 LMRGs were gained through Reactome and the Kyoto Encyclopedia of Genes and Genomes (KEGG) repository [5].

Identification of AR-related genes (ARRGs)

To gain ARRGs in GSE75011, weighted gene coexpression network analysis (WGCNA) was performed. First, the samples were clustered in order to remove outliers. Thereafter, the determination of soft threshold (β) was performed. Modules were segmented via dynamic tree cutting based on optimal β. Correlations were analyzed between modules and AR. The genes of the highest relevance module with |gene significance (GS)| > 0.3, |module membership (MM)| > 0.6, and $P < 0.05$ were defined as ARRGs [10].

Screening and functional analysis of lipid metabolism-related differentially expressed genes (LMR DEGs)

First, sample normalization on the GSE75011 dataset was performed via "limma" R package (version 3.48.3). The mRNA expression levels between control and AR groups in the GSE75011 dataset were contrasted via the "limma" R package (version 3.48.3) ($P < 0.05$) [11]. DEGs and LMRGs were taken to intersect to get LMR DEGs. Subsequently, enrichment analysis for LMR DEGs via Metascape database ($P < 0.05$) [12]. In addition, the online database WebGestalt was used to study the isease Ontology (DO) function of LMR DEGs.

Creation of protein–protein interaction (PPI) networks of LMR DEGs and screening of hub genes

PPIs of LMR DEGs were created via Search Tool for the Retrieval of Interacting Genes (STRING). Subsequently, Cytoscape was utilized to visualize PPIs, and hub genes were obtained by intersecting the top 30 genes calculated by stress, radiality, closeness, and edge percolated component (EPC).

Screening of diagnostic genes

First, candidate genes were obtained by intersecting hub genes with ARRGs, and enrichment analysis were applied on them. Second, receiver operating characteristic (ROC) curves of the candidate genes were mapped via "pROC" R package (version

1.18.0) in the GSE75011 and GSE46171[13]. Candidate genes with area under the curve (AUC) ≥ 0.7 were regarded diagnostic genes. A nomogram was constructed with hub genes, and a calibration curve of the nomogram was drawn to verify its validity. The diagnostic worth of age, sex, and time point was assessed in GSE46171 via ROC curves.

Immune analysis

The single-set gene set enrichment analysis (ssGSEA) algorithm was utilized to assess infiltrating richness of immune cells between AR and normal groups in the training set. Differences of the control and AR groups were compared by the Wilcox test. In addition, relevance was analyzed via Spearman algorithm between diagnostic genes and differential immune cells.

Analysis of clinical correlation

Relevance between diagnostic genes and clinical characteristics (age, sex, time point) was analyzed using Pearson in the "corrplot" R package [14].

Gene Set Enrichment Analysis (GSEA) of diagnostic genes

On the basis of the median value of the diagnostic genes expression, the samples of GSE75011 were grouped into high and low expression groups. All genes in two expression groups were performed GSEA with |normalized enrichment score (NES)| > 1, nominal (NOM) *P* value < 0.05, and *q* < 0.25 [15].

Expression profiles of diagnostic genes in external validation datasets

To further demonstrate the reliability of the results above, expression levels of the diagnostic genes between AR and control samples were compared in the GSE75011 and GSE46171 datasets for external validation.

Patients and tissue preparation

Ten AR patients and ten patients without AR or significant underlying disease were selected from people visiting to Shanghai Changzheng Hospital. There were no marked variation in sex and age between the groups (Table 1). Blood samples were acquired from these patients with informed consent and carried out reverse transcription-polymerase chain reaction (RT–qPCR). This Medical Ethics Committee of Shanghai Changzheng Hospital endorsed this study.

Table 1 Basic information of the patients

Group	Sex		Age (mean ± STD)
	Male	Female	
AR	6	4	29.9±5.705
Control	5	5	28.4±3.098

STD, standard deviation

RT–qPCR

Firstly, we conducted the total RNA extraction utilizing TRIzol (Ambion, Austin, USA). Then, reverse transcription of total RNA to cDNA was made via First-strand-cDNA-synthesis-kit (Servicebio, Wuhan, China). RT–qPCR was made utilizing the 2xUniversal Blue SYBR Green qPCR Master Mix (Servicebio, Wuhan, China). Specific experimental steps were carried out on the basis of instructions. The primer sequences were showcased in Additional file 1. Internal reference gene was GAPDH. The $2^{-\Delta\Delta Ct}$ method was utilized to calculate the expression of diagnostic genes [16]. Levels of expression of diagnostic genes between the control and AR groups were compared by the T test.

Statistical analysis

Statistical analysis was carried out through GraphPad Prism 5 and R software (version 4.2.0). $P < 0.05$ represented a significant difference. Differences between groups were analyzed via the Wilcoxon test.

Results

Acquisition of ARRGs

To identify ARRGs, WGCNA was performed with the GSE75011 dataset. Sample clustering analysis showed no outliers in the dataset (Figure 1A). The β was 4 (Figure 1B), and each gene module contained a minimum of 100 genes. Three modules were eventually identified, each with a unique color (Figure 1C-D). The blue module

correlated markedly with AR (cor = -0.35, P = 0.03) (Figure 1D). Finally, 66 ARRGs were gained and utilized for further analysis (Figure 1E).

Acquisition and functional enrichment of LMR DEGs

The 25 samples were standardized for the GSE75011 dataset and are presented as box plots in Figure 2A-B. The volcano plot and heatmap show 1621 DEGs between the AR and control groups, including 810 upregulated and 811 downregulated genes (Figure 2C-D). A total of 73 LMR DEGs (Additional file 2) were obtained by Venn analysis with LMRGs (810 genes) and DEGs (1621 genes), with a significant difference detected based on a heatmap (Figure 2E-F). Enrichment analysis of the 73 LMR DEGs by Metascape showed a total of 334 functional pathways (Figure 2G-H) to be related to the LMR DEGs, such as metabolism of lipids, lipid biosynthetic process, and sterol regulatory element-binding protein (SREBP) signaling. DO enrichment results showed that the LMR DEGs are significantly associated with 10 diseases, namely, xanthomatosis, increased serum pyruvate, decreased high-density lipoprotein, hypoalphalipoproteinemia, myoglobinuria, insulin-resistant diabetes, neonatal death, thin skin, myalgia and cardiomegaly (Figure 2I).

Acquisition of hub genes

The PPI network was created for LMR DEGs. As illustrated in Figure 3A-B, SREBF1 interacts with multiple proteins, such as LPIN1, GPAM, and MED1. To identify the most important genes, the 22 genes common to the 4 algorithms were used as hub

genes (Figure 3C), and a PPI network of hub genes was created (Figure 3D). The results showed that GPAM interacts with 7 genes, namely, PPARG, NFYA, SREBF1, ACSL3, LPIN1, HMGCS1 and AACS.

Acquisition of diagnostic genes

Four candidate genes associated with diagnosis of AR were obtained by 66 ARRGs with 22 hub genes taking intersections: LPCAT1, SREBF1, SMARCD3, and SGPP1 (Figure 4A). The four candidate genes were involved in 133 GO items, including 114 GO BP, 9 GO CC and 10 GO MF, such as retina development in camera-type eye, npBAF complex, and transcription coregulator binding (Figure 4B).

The diagnostic value of four candidate genes was assessed via ROC curve in GSE75011 and GSE46171. The AUC values for the three genes (LPCAT1, SMARCD3, and SGPP1) were greater than 0.7 in both datasets, suggesting that the three genes have diagnostic value for AR (Figure 4C-D). The AUCs for age, sex and time point were 0.4492, 0.4839, and 0.7166, respectively, in GSE46171, revealing that sex might be a diagnostic factor for AR (Figure 4E).

Finally, the nomograms were created containing the three diagnostic genes in GSE75011 and GSE46171 (Figure 5A-B), and the AUC values in both datasets were above 0.6 (Figure 5C-D). The results demonstrated that the nomogram has good prediction ability for AR .

Immuno-infiltration analysis in AR and control groups

Analysis of the percentage of immune cells by ssGESA in all samples showed the highest for T cells (Figure 6A). Differences in infiltrating immune cells between the AR and control groups were illustrated by a violin plot (Figure 6B). The results suggested that infiltration of regulatory T cells (TRegs) and T follicular helper cells (TFHs) was markedly lower in the AR group. There was significant relevance between SMARCD3 and TFHs. However, neither LPCAT1 nor SGPP1 correlated with differential immune cells (TRegs and TFHs); therefore, SMARCD3 was selected for further analysis (Figure 6C-E).

Correlation analysis of clinical features, enrichment analysis and infiltration analysis of SMARCD3

Pearson correlation analysis demonstrated that SMARCD3 was significantly associated with clinical characteristics (age and sex) (Figure 7A-C). Then, GSEA for SMARCD3 was performed, revealing 256 GO enrichment (Additional file 3) and 33 KEGG (Additional file 4) pathways (Figure 7D-E). Overall, SMARCD3 was involved in immune-related pathways, for instance, the B-cell receptor signaling pathway and T-cell receptor signaling pathway. Four immune cells displayed marked variations between the high and low expression groups, namely, macrophages, T helper cells, Tcm, and TFH cells, reflecting the strong relevance between SMARCD3 and the immune microenvironment (Figure 7F).

mRNA levels of diagnostic genes

The significant differences in expression of SGPP1, LPCAT1 and SMARCD3 between control and AR in GSE75011 and GSE46171 were clearly observed via visualized data (Figure 8A-B). Moreover, the changes of the three genes expression were consistent in blood and nasal mucosal tissues, suggesting that these three genes are of high diagnostic value.

To verify diagnostic gene expression, we collected blood samples to assess mRNA expression levels of three prognostic genes via RT-qPCR. The expression trends of LPCAT1 and SMARCD3 were consistent with public databases, and the expression was lower in AR group (Figure 9A-B). However, SGPP1 exhibited the opposite trend compared to the results of public database, possibly due to different experimental designs or analysis methods (Figure 9C).

Discussion

AR is an airway allergic disease with a high incidence, affecting billions of people in the world. Nevertheless, the effect of current therapies for AR is unsatisfactory due to its complex pathogenesis. LMRGs are involved in the maintenance of systemic physiology and play an important role in diverse diseases, especially in malignant tumors. Moreover, lipid-related inflammatory mediators such as prostaglandins and leukotrienes have been implicated in AR pathogenesis. To our knowledge, this is the first study to identify and analyze LMRGs in AR.

In this study, three key LMRGs most associated with AR, i.e., LPCAT1, SGPP1, and SMARCD3, were identified, all of which are protein-coding genes. As one of the lysophosphatidylcholine acyltransferase (LPCAT) family, the LPCAT1 protein is an enzyme essential for phosphatidylcholine metabolism and regulation of phosphatidylcholine composition [17]. LPCAT1 is also used in multiple tumors' prognosis, such as colorectal cancer, breast cancer, and hepatocellular carcinoma[18-20]. Little is known about LPCAT1 in allergic diseases. One study reported that LPCAT1 downregulates eosinophilic inflammation in asthmatic mice [21]. In the current study, LPCAT1 was significantly lower in AR blood samples, consistent with published results, suggesting that it may be essential for AR pathogenesis. SGPP1 can catalyze degradation of S1P, who can regulate diverse biological processes, as a bioactive sphingolipid metabolite [22]. SGPP1 is considered to be closely related to several tumors, especially regarding chemoresistance and radioresistance [23, 24]. There are currently no reports about the function of SGPP1 in allergic diseases, and the results in the current study are the first to show significant downregulation of SGPP1 in both blood and nasal mucosa samples in AR patients; conversely, RT–qPCR using blood samples showed the opposite result, possibly due to different experimental designs or analysis methods. Thus, the effect of SGPP1 in AR is still unclear. SMARCD3 is a chromatin-remodeling factor and a member of the SWI/SNF family, which present helicase and ATPase activities and are crucial in the transcription process of certain genes. Its related pathways include the circadian clock and transcriptional activation of mitochondrial biogenesis [25]. SMARCD3 was found

to be downregulated in AR patients in this study, but how it participates in disease processes remains to be explored.

Immuno-infiltration analysis refers to studying the composition and quantification of immune cells in diseases. In this study, T follicular helper cells (TFHs) were extremely significantly reduced in the AR group. TFHs are $CD4^+$ T cells that specialize in helping B cells and are involved in a wide range of diseases. An increasing number of theories have concluded that the antigen-related IgE response depends on more TFHs than Th2 cells [26, 27]. There are few reports about TFH and SMARCD3. A microarray model system identified that the SMARCD3 gene is upregulated in T-cell acute lymphoblastic leukemia [28]. In this study, only SMARCD3 correlated with differential immune cells (TRegs and TFHs), and TFHs and SMARCD3 were downregulated simultaneously in AR patients. Hence, it is hypothesized that SMARCD3 participates in the differentiation of T cells.

GSEA was performed to further investigate the role of SMARCD3 in AR, the results of which showed significant enrichment in the adipocytokine signaling pathway, B-cell receptor signaling pathway, and chemokine signaling pathway, among others. The adipocytokine signaling pathway refers to a series of cascade events via autocrine or paracrine adipocytokines, such as leptin and adiponectin, by adipocytes in the body [29, 30]. This pathway is not only crucial for obesity, insulin resistance, and type II diabetes mellitus but also plays an important role in inflammation and allergic diseases. Dysregulation of pulmonary adipocytokine/insulin signaling caused by early-onset obesity has been proven to induce asthma-like disease in mice [31]. The

leptin/osteopontin axis promotes Th2 inflammation and Th17 responses in AR through the NF-κB, MAPK, JNK pathway and β3 integrin [32, 33]. Signaling through the B-cell receptor (BCR) is crucial for antigen recognition and subsequent biological effects, including B-cell activation, proliferation, and differentiation, which ensure host defense [34]. One study demonstrated that the BCR signaling pathway was significantly enriched among differentially expressed vesicle miRNAs in AR patient nasal mucus, consistent with the findings in the current study and further elucidating the importance of the BCR signaling pathway in AR development [35]. Chemokines are small molecule-scale cytokines that recruit leukocyte subsets under steady-state and pathological conditions; signaling pathways are activated by their binding to receptors on the cell surface and are involved in chronic inflammatory and autoimmune diseases. Multiple studies have shown that knockdown of the chemokine receptor CCR3 reduces eosinophilic inflammation and the Th2 immune response in AR [36-38]. In summary, our findings are in accordance with all of the above studies.

Comparisons with other studies and contribution of the current work to existing knowledge

To the best of our knowledge, exploration of AR based on GSE75011 and GSE46171 has mainly targeted key genes differentially expressed between AR and control samples [39-42]. In the current study, the biological significance of lipid metabolism in AR was first systematically explored at the genetic level through these datasets. Moreover, correlation between SMARCD3 expression and immune cell infiltration

was investigated to elucidate the underlying role of immune-related treatment targeting the SMARCD3 gene in exploration of AR development.

Study strengths and limitations

Three key LMRGs with high diagnostic values for AR were identified and analyzed for the first time based on bioinformatics analysis of AR-related expression datasets. However, the limitations of this study cannot be ignored. First, small sample sizes and small datasets of AR may have introduced bias. Second, the mechanisms of these genes in AR development have not been clearly elucidated. Deeper research may be needed for the possibility of clinical use in the future.

Conclusions

In summary, this is the first bioinformatics analysis of LMRGs in AR, and three key genes (LPCAT1, SGPP1 and SMARCD3) with high diagnostic value for AR were identified. A highly accurate nomogram was constructed to validate the clinical value of the gene-based diagnostic model. In addition, two of these genes were confirmed by clinical validation and are considered potential treatment targets. In particular, the correlation of SMARCD3 expression and immune cell infiltration was helpful to reveal future research directions of immune-related treatment targeting the SMARCD3 gene in AR.

References

1. Brozek JL, Bousquet J, Baena-Cagnani CE, Bonini S, Canonica GW, Casale TB, et al. Allergic Rhinitis and its Impact on Asthma (ARIA) guidelines: 2010 revision. J Allergy Clin Immunol.

2010;126(3):466-76.
2. Wheatley LM, Togias A. Clinical practice. Allergic rhinitis. N Engl J Med. 2015;372(5):456-63.
3. Seidman MD, Gurgel RK, Lin SY, Schwartz SR, Baroody FM, Bonner JR, et al. Clinical practice guideline: Allergic rhinitis. Otolaryngol Head Neck Surg. 2015;152(1 Suppl):S1-43.
4. Olzmann JA, Carvalho P. Dynamics and functions of lipid droplets. Nat Rev Mol Cell Biol. 2019;20(3):137-55.
5. Li W, Ding Z, Zhang H, Shi Q, Wang D, Zhang S, et al. The Roles of Blood Lipid-Metabolism Genes in Immune Infiltration Could Promote the Development of IDD. Front Cell Dev Biol. 2022;10:844395.
6. Merino Salvador M, Gomez de Cedron M, Moreno Rubio J, Falagan Martinez S, Sanchez Martinez R, Casado E, et al. Lipid metabolism and lung cancer. Crit Rev Oncol Hematol. 2017;112:31-40.
7. Lee K, Lee SH, Kim TH. The Biology of Prostaglandins and Their Role as a Target for Allergic Airway Disease Therapy. Int J Mol Sci. 2020;21(5).
8. Yokomizo T, Nakamura M, Shimizu T. Leukotriene receptors as potential therapeutic targets. J Clin Invest. 2018;128(7):2691-701.
9. Tomazic PV, Birner-Gruenberger R, Leitner A, Darnhofer B, Spoerk S, Lang-Loidolt D. Apolipoproteins have a potential role in nasal mucus of allergic rhinitis patients: a proteomic study. Laryngoscope. 2015;125(3):E91-6.
10. Langfelder P, Horvath S. WGCNA: an R package for weighted correlation network analysis. BMC Bioinformatics. 2008;9:559.
11. Ritchie ME, Phipson B, Wu D, Hu Y, Law CW, Shi W, et al. limma powers differential expression analyses for RNA-sequencing and microarray studies. Nucleic Acids Res. 2015;43(7):e47.
12. Wu T, Hu E, Xu S, Chen M, Guo P, Dai Z, et al. clusterProfiler 4.0: A universal enrichment tool for interpreting omics data. Innovation (Camb). 2021;2(3):100141.
13. Robin X, Turck N, Hainard A, Tiberti N, Lisacek F, Sanchez JC, et al. pROC: an open-source package for R and S+ to analyze and compare ROC curves. BMC Bioinformatics. 2011;12:77.
14. Hauben M, Hung E, Hsieh WY. An exploratory factor analysis of the spontaneous reporting of severe cutaneous adverse reactions. Ther Adv Drug Saf. 2017;8(1):4-16.
15. Ledley FD, Grenett HE, McGinnis-Shelnutt M, Woo SL. Retroviral-mediated gene transfer of human phenylalanine hydroxylase into NIH 3T3 and hepatoma cells. Proc Natl Acad Sci U S A. 1986;83(2):409-13.
16. Livak KJ, Schmittgen TD. Analysis of relative gene expression data using real-time quantitative PCR and the 2(-Delta Delta C(T)) Method. Methods. 2001;25(4):402-8.
17. Harayama T, Eto M, Shindou H, Kita Y, Otsubo E, Hishikawa D, et al. Lysophospholipid acyltransferases mediate phosphatidylcholine diversification to achieve the physical properties required in vivo. Cell Metab. 2014;20(2):295-305.
18. Lebok P, von Hassel A, Meiners J, Hube-Magg C, Simon R, Hoflmayer D, et al. Up-regulation of lysophosphatidylcholine acyltransferase 1 (LPCAT1) is linked to poor prognosis in breast cancer. Aging (Albany NY). 2019;11(18):7796-804.
19. Mansilla F, da Costa KA, Wang S, Kruhoffer M, Lewin TM, Orntoft TF, et al. Lysophosphatidylcholine acyltransferase 1 (LPCAT1) overexpression in human colorectal cancer. J Mol Med (Berl). 2009;87(1):85-97.
20. Li L, Wang X, Ding Y, Hui N, Su B, Yang M. LPCAT1 acts as an independent prognostic biomarker correlated with immune infiltration in hepatocellular carcinoma. Eur J Med Res. 2022;27(1):216.
21. Cheng S, Chen H, Wang A, Xie M, Xie J, Osanai K, et al. Lentiviral vector-mediated delivery of

lysophosphatidylcholine acyltransferase 1 attenuates airway inflammation in ovalbumin-induced allergic asthmatic mice. Asian Pac J Allergy Immunol. 2015;33(4):320-9.

22. Le Stunff H, Giussani P, Maceyka M, Lepine S, Milstien S, Spiegel S. Recycling of sphingosine is regulated by the concerted actions of sphingosine-1-phosphate phosphohydrolase 1 and sphingosine kinase 2. J Biol Chem. 2007;282(47):34372-80.

23. Huang X, Taeb S, Jahangiri S, Emmenegger U, Tran E, Bruce J, et al. miRNA-95 mediates radioresistance in tumors by targeting the sphingolipid phosphatase SGPP1. Cancer Res. 2013;73(23):6972-86.

24. Zhang B, Gao S, Bao Z, Pan C, Tian Q, Tang Q. MicroRNA-656-3p inhibits colorectal cancer cell migration, invasion, and chemo-resistance by targeting sphingosine-1-phosphate phosphatase 1. Bioengineered. 2022;13(2):3810-26.

25. He A, Kong SW, Ma Q, Pu WT. Co-occupancy by multiple cardiac transcription factors identifies transcriptional enhancers active in heart. Proc Natl Acad Sci U S A. 2011;108(14):5632-7.

26. Crotty S. T Follicular Helper Cell Biology: A Decade of Discovery and Diseases. Immunity. 2019;50(5):1132-48.

27. Kamekura R, Shigehara K, Miyajima S, Jitsukawa S, Kawata K, Yamashita K, et al. Alteration of circulating type 2 follicular helper T cells and regulatory B cells underlies the comorbid association of allergic rhinitis with bronchial asthma. Clin Immunol. 2015;158(2):204-11.

28. Hoffmann K, Dixon DN, Greene WK, Ford J, Taplin R, Kees UR. A microarray model system identifies potential new target genes of the proto-oncogene HOX11. Genes Chromosomes Cancer. 2004;41(4):309-20.

29. Galic S, Oakhill JS, Steinberg GR. Adipose tissue as an endocrine organ. Mol Cell Endocrinol. 2010;316(2):129-39.

30. Achari AE, Jain SK. Adiponectin, a Therapeutic Target for Obesity, Diabetes, and Endothelial Dysfunction. Int J Mol Sci. 2017;18(6).

31. Dinger K, Kasper P, Hucklenbruch-Rother E, Vohlen C, Jobst E, Janoschek R, et al. Early-onset obesity dysregulates pulmonary adipocytokine/insulin signaling and induces asthma-like disease in mice. Sci Rep. 2016;6:24168.

32. Liu W, Zeng Q, Zhou L, Li Y, Chen Y, Luo R. Leptin/osteopontin axis contributes to enhanced T helper 17 type responses in allergic rhinitis. Pediatr Allergy Immunol. 2018;29(6):622-9.

33. Zeng Q, Luo X, Han M, Liu W, Li H. Leptin/Osteopontin Axis Regulated Type 2T Helper Cell Response in Allergic Rhinitis with Obesity. EBioMedicine. 2018;32:43-9.

34. Tanaka S, Baba Y. B Cell Receptor Signaling. Adv Exp Med Biol. 2020;1254:23-36.

35. Wu G, Yang G, Zhang R, Xu G, Zhang L, Wen W, et al. Altered microRNA Expression Profiles of Extracellular Vesicles in Nasal Mucus From Patients With Allergic Rhinitis. Allergy Asthma Immunol Res. 2015;7(5):449-57.

36. Fortin M, Wagner JG, Brault J, Harkema JR, Renzi PM, Paquet L, et al. Spatial and temporal expression of CCR3 and the common beta chain of the IL-3, IL-5 and GM-CSF receptor in the nasal epithelium and lymphoid tissues in a rat model of allergic rhinitis. Cytokine. 2010;52(3):194-202.

37. Dai M, Zhu X, Yu J, Yuan J, Zhu Y, Bao Y, et al. CCR3 gene knockout in bone marrow cells ameliorates combined allergic rhinitis and asthma syndrome (CARAS) by reducing airway inflammatory cell infiltration and Th2 cytokines expression in mice model. Int Immunopharmacol. 2022;104:108509.

38. Yuan J, Liu Y, Yu J, Dai M, Zhu Y, Bao Y, et al. Gene knockdown of CCR3 reduces eosinophilic

inflammation and the Th2 immune response by inhibiting the PI3K/AKT pathway in allergic rhinitis mice. Sci Rep. 2022;12(1):5411.
39. Chang WC, Xu Y. [Integrated bioinformatics analysis of key genes in allergic rhinitis]. Zhonghua Er Bi Yan Hou Tou Jing Wai Ke Za Zhi. 2020;55(5):458-64.
40. Yan Z, Liu L, Jiao L, Wen X, Liu J, Wang N. Bioinformatics Analysis and Identification of Underlying Biomarkers Potentially Linking Allergic Rhinitis and Asthma. Med Sci Monit. 2020;26:e924934.
41. Hao Y, Wang B, Zhao J, Wang P, Zhao Y, Wang X, et al. Identification of gene biomarkers with expression profiles in patients with allergic rhinitis. Allergy Asthma Clin Immunol. 2022;18(1):20.
42. Ma Y, Liu C, Xi G, Guan Y, Tang Y, Zhang J, et al. Bioinformatic Analysis and Cellular Assays Identify Substance P Influencing Th17/Treg Differentiation via the MyD88 Pathway as a Potential Contributor to the Progression of Asthma and Allergic Rhinitis. Dis Markers. 2022;2022:3843954.

13% Overall Similarity

Top sources found in the following databases:

- 9% Internet database
- 10% Publications database
- Crossref database
- Crossref Posted Content database
- 0% Submitted Works database

TOP SOURCES

The sources with the highest number of matches within the submission. Overlapping sources will not be displayed.

#	Source	Database	Similarity
1	frontiersin.org	Internet	2%
2	hindawi.com	Internet	1%
3	portlandpress.com	Internet	<1%
4	Xinming Chen, Zheng Zhu, Xiaoling Li, Xinyue Yao, Lianxiang Luo. "The ...	Crossref	<1%
5	academic.oup.com	Internet	<1%
6	Jinyou Li, Qiang Li, Zhenyu Su, Qi Sun, Yong Zhao, Tienan Feng, Jiayua...	Crossref	<1%
7	Xiaokai Li, Qiuming Su, Wang Li, Xibing Zhang, Jianghua Ran. "Analysis...	Crossref	<1%
8	assets.researchsquare.com	Internet	<1%

9	dovepress.com<br>Internet	<1%
10	Yuxin Ran, Jie He, Wei Peng, Zheng Liu, Youwen Mei, Yunqian Zhou, Na...<br>Crossref	<1%
11	researchsquare.com<br>Internet	<1%
12	Bruce M. Prenner, Niran J. Amar, Frank C. Hampel, Cynthia F. Caracta, ...<br>Crossref	<1%
13	static-site-aging-prod2.impactaging.com<br>Internet	<1%
14	mdpi.com<br>Internet	<1%
15	Cruceanu, Cristiana. "Investigation of Genetic and Molecular Susceptib...<br>Publication	<1%
16	Qingqing Zhao, Shifei Zhang, Xiaolong Chang, Dan Wang, Qinglong Ai, ...<br>Crossref posted content	<1%
17	conpath.dna-chip.co.jp<br>Internet	<1%
18	Wu, Geping, Guanghai Yang, Ruxin Zhang, Guangyin Xu, Ling Zhang, W...<br>Crossref	<1%
19	hereditasjournal.biomedcentral.com<br>Internet	<1%
20	jeccr.biomedcentral.com<br>Internet	<1%

21	jove.com<br>Internet	<1%
22	medrxiv.org<br>Internet	<1%
23	nature.com<br>Internet	<1%
24	Basavaraj Vastrad, Chanabasayya Vastrad. "The Identification of Key G...<br>Crossref posted content	<1%
25	Geping Wu, Guanghai Yang, Ruxin Zhang, Guangyin Xu, Ling Zhang, Wu...<br>Crossref	<1%
26	Kotaro Matsumoto, Katsuya Suzuki, Hiroto Yoshida, Mayu Magi, Yuko ...<br>Crossref	<1%
27	Weihong Chen, Jianzhi Deng, Yuehan Zhou. "The Construction of a Nov...<br>Crossref posted content	<1%
28	Wenlong Liu, Qingxiang Zeng, Lifeng Zhou, Yan Li, Yanqiu Chen, RenZh...<br>Crossref	<1%
29	Xueping Qi, Hedi Zhuo, Yanjie Wang, Fanggang Ren, Jinmei Xue, Yunfa...<br>Crossref	<1%
30	cancerdiscovery.aacrjournals.org<br>Internet	<1%
31	medscimonit.com<br>Internet	<1%
32	pure.eur.nl<br>Internet	<1%

33	spandidos-publications.com<br>Internet	<1%
34	Edwin Chen, Xiaoyong Huang, Yanzhen Zheng, You-Jun Li, Alden Ches...<br>Crossref	<1%
35	Xi Luo, Qingxiang Zeng, Shengbao Yan, Wenlong Liu, Renzhong Luo. "...<br>Crossref	<1%
36	Gabija Drazdauskaitė, Janice A. Layhadi, Mohamed H. Shamji. "Mecha...<br>Crossref	<1%
